# Supplementary material for: Unique information from common diffusion MRI models about white-matter differences across the human adult lifespan
Source: Imaging Neurosci (Camb). 2023 Dec 21;1:imag-1-00051. doi: 10.1162/imag_a_00051 (PMC12327084; doi:10.1162/imag_a_00051)
Supplement: Supplementary Material [file imag_a_00051-supp.pdf]

Unique information from common diffusion MRI models about white-matter differences across the human adult lifespan

SUPPLEMENTARY MATERIAL

Rafael Neto Henriques[1,*], Richard Henson[2,3], Cam-CAN[4] & Marta Morgado Correia[2]

[1] Champalimaud Research, Champalimaud Foundation, Portugal.

[2] MRC Cognition and Brain Sciences Unit, University of Cambridge, United Kingdom.

[3] Department of Psychiatry, University of Cambridge, United Kingdom.

[4] Cambridge Centre for Ageing and Neuroscience (Cam-CAN), University of Cambridge, United Kingdom

***** corresponding author: rafael.henriques@neuro.fchampalimaud.org

Appendix A. Quantification of images corrupted by motion-related artefacts

The two phases used to quantify diffusion-weighted volumes corrupted by motion artefacts are described in this section.

Phase 1: Head motion during data acquisition not only induces volume misalignments but also signal loss due to abrupt tissue displacement (e.g., Tournier et al., 2011). For axial acquisitions, as is the case for the data acquired on the Cam-CAN project, this can be qualitatively observed along sagittal slices in the form of single slice signal loss or in the form of a "striping" pattern of intensities (Tournier et al., 2011). For the present study, this type of artefact is quantified by convolution of each sagittal slice of all diffusion-weighted volumes with the kernel shown in step 1, supplementary Fig. S1. Supplementary Fig. S1 shows the effect of the kernel convolution on images that are (panel A) and are not (panel B) corrupted by motion artefacts. As supplementary Fig. S1 shows, resulting images present greater intensities if raw images were corrupted by this type of artefact (step 2 in supplementary Fig. S1). Voxel intensities were then added to generate a single value for each volume (step 3 in supplementary Fig. S1). This measure is designated here as the apparent "stripe" index (*aSI*), since its magnitude will not only depend on the amount of motion artefacts, but also on morphological characteristics of the brain of each subject (e.g. larger brains have more brain voxels) and on the diffusion-weighted parameters (e.g. higher tissue intensities on lower b-values are intrinsically associated with higher contrasts between tissue intensity and signal loss and thus larger values of *aSI*). To remove the latter two dependencies, *aSI* values for the same subject and b-value are grouped and normalized by its group minimum (step 4 in supplementary Fig. S1), resulting in values equal or larger than 1, which we defined as the corrected stripe index *cSI*.

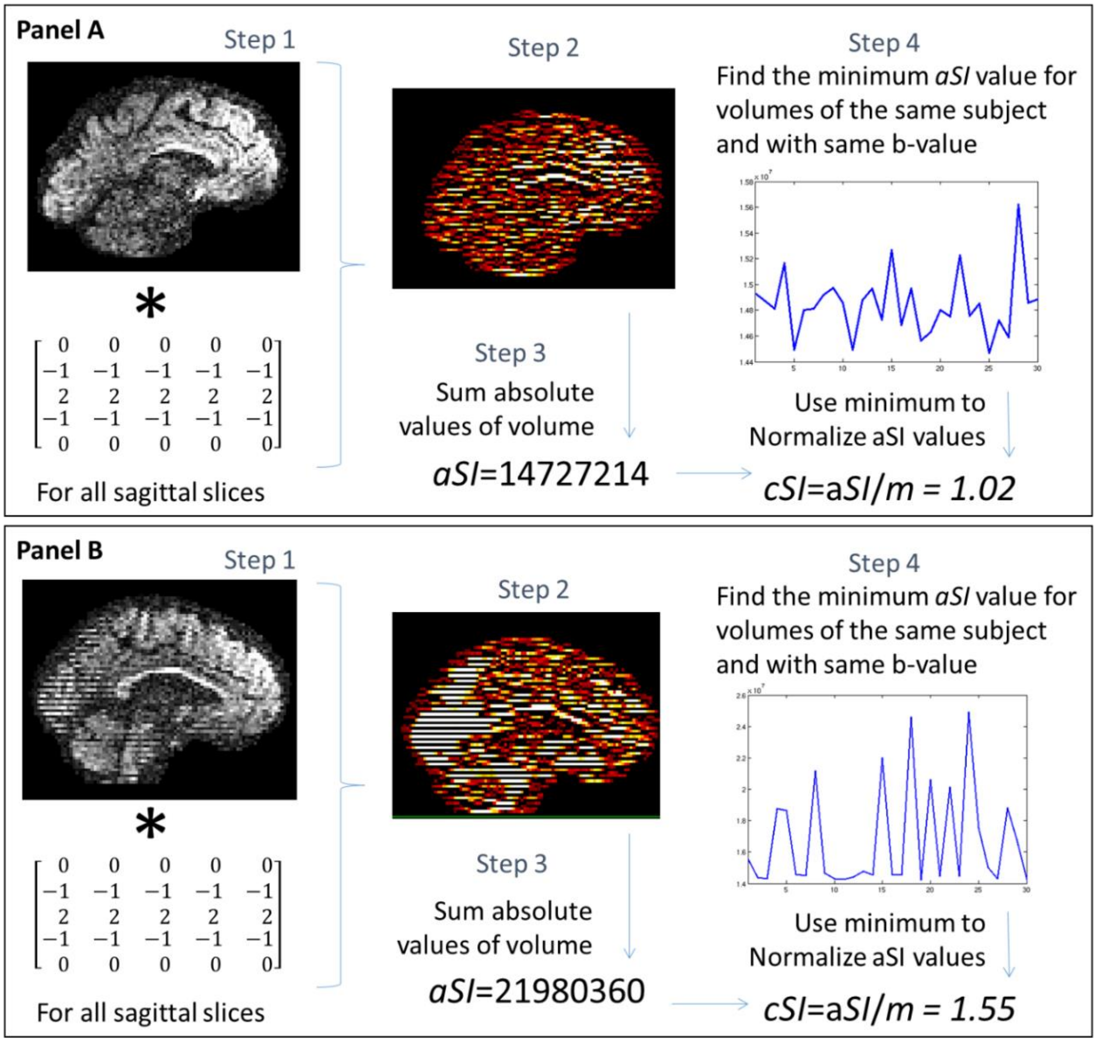

Supplementary Fig. S1 – First phase of the procedure to quantify the artefacts induced by abrupt subject motion during data acquisition. On panel A the procedure is illustrated for a volume not corrupted by motion artefacts, while panel B for a volume that visually revealed evident stripe artefacts.

Phase 2: From empirical observations, volumes with cSI larger than 1.25 were shown to be highly corrupted with stripe artefacts, while non-problematic volumes were shown to have cSI lower than 1.15. For values between 1.15 and 1.25, some volumes were visually identified as corrupted by signal loss. This was observed only for volumes acquired along specific diffusion gradient directions, indicating that the dependency on the diffusion gradient is still present in the normalised parameter *cSI*. To remove these extra dependencies, a further correction is applied to the *cSI* values using the procedure illustrated in supplementary Fig. S2. For all subjects, *cSI* values are organized in the order that the associated b-values and diffusion gradients directions were acquired (step 1 in supplementary Fig. S2); the expected *cSI* profile

along the diffusion gradient directions is computed from the mean values of *cSI* of non-problematic subjects, i.e. subjects that have any volume with *cSI* larger than 1.25 were removed (step 2 in supplementary Fig. S2); all individual curves of *cSI* measures are then detrended by the expected *cSI* profile (step 3 in supplementary Fig. S2). This procedure gives a value different from zero as its associated *cSI* value deviates from the expected *cSI* variance profile. Here, detrended "stripe" indexes will be referred to as the full corrected stripe index *fSI*. From visual inspections, this measure was able to distinguish volumes that were corrupted by motion artefacts from the ones that were not by setting a threshold value of 0.1.

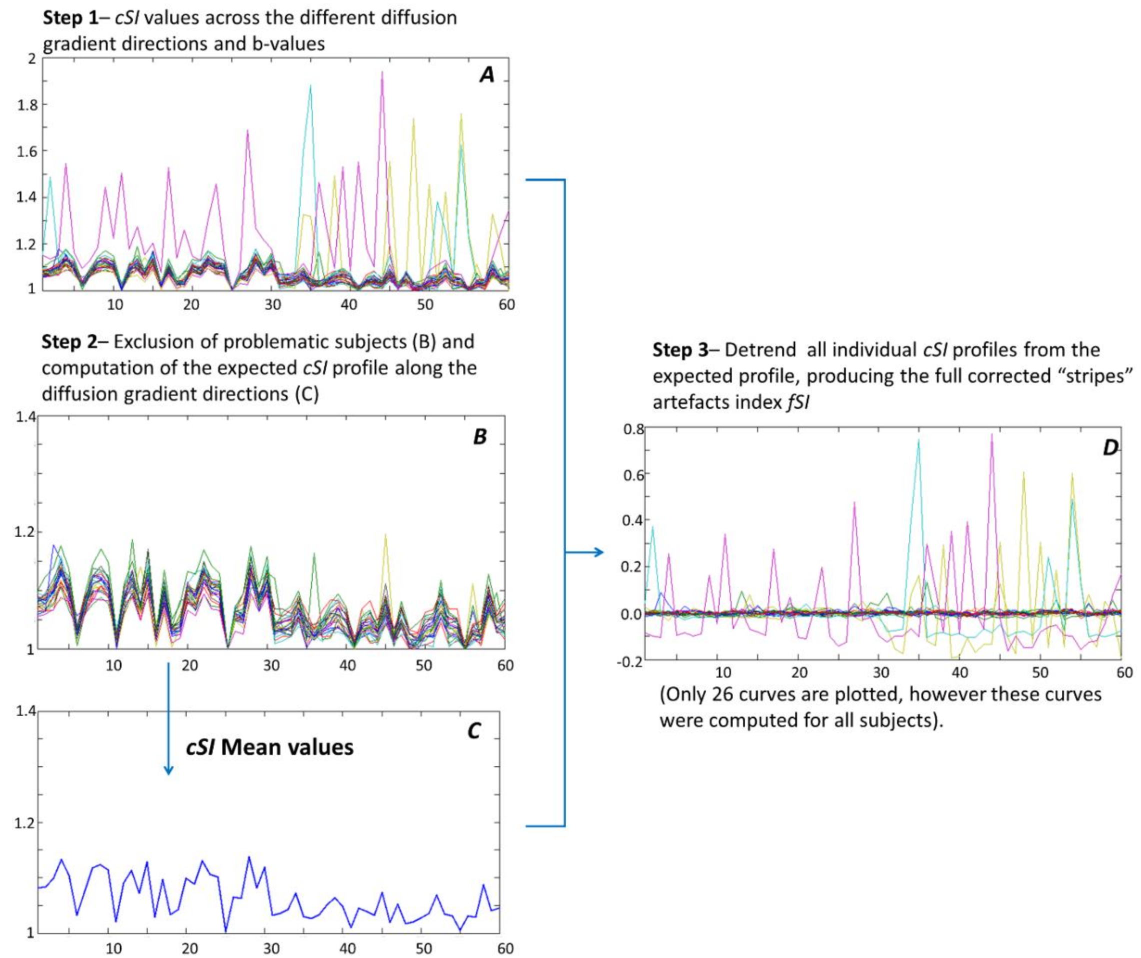

Supplementary Fig. S2 –Second phase of the procedure to quantify the artefacts induced by abrupt subject motion during data acquisition. (A) cSI values across the different diffusion gradient directions and b-values are plotted together. (B) cSI values for subject that showed a cSI value larger than 1.25 are removed; (C) cSI values for selected subjects are averaged; (D) cSI profiles from individual subjects are detrended from the averaged profiles obtaining the final fSI values.

Appendix B. Correcting for motion-induced image misalignments

In this study, motion misalignments are corrected by registering each diffusion-weighted volume to a simulated template, allowing for the inherent contrast differences expected from dMRI acquisitions at different b-values and diffusion gradient directions.

The steps to generate the specific templates are summarized in supplementary Fig. S3. Firstly, select the data acquired with b-value = 1000s/mm^2, avoiding larger amount of motion on the higher b-value data (step 1) to generate a first version of the templates. This first version is generated using the predicted signals of a diffusion tensor model fit to the selected b-value = 1000s/mm^2 data (step 2). To reduce the impact of possible artefacts on the low b-value regime, the data is smoothed (Gaussian kernel with FWHM = 1.25 × voxel size) and non-brain voxels removed before DTI fitting. The preliminary version of the templates is used to correct misalignments from the initial b-value data (step 3). The final version of the templates is then produced as the signal prediction of the diffusion tensor model re-fitted to the aligned data. These final versions of the templates are then produced for all b-values and gradient directions of the original dataset (step 4).

After the generation of templates, each original diffusion-weighted volume is aligned to its corresponding template using six degrees of freedom registration with the residual sum of squares minimization cost function. To preserve the correct association between the diffusion acquisition protocol and the image contrast, the motion parameters computed from each image registration are used to rotate the corresponding diffusion-weighted gradient direction.

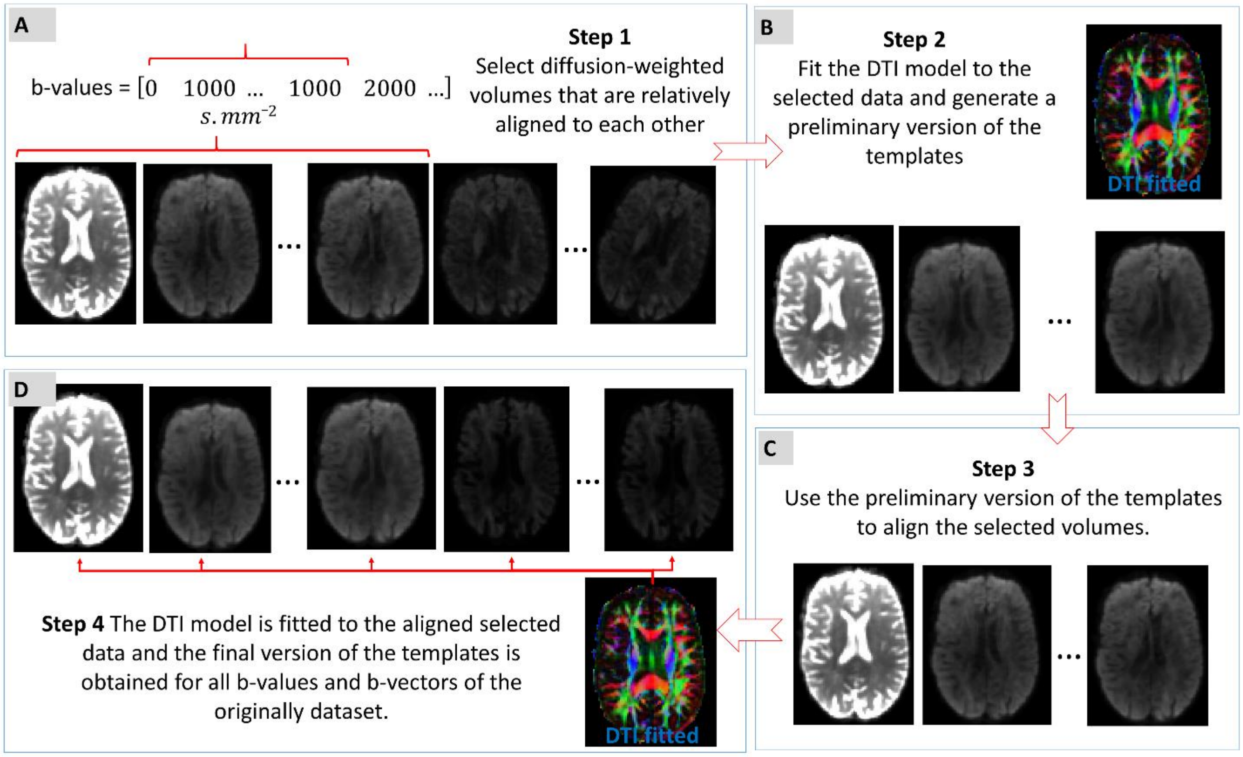

Supplementary Fig. S3 – Procedure to generate templates specific to the diffusion gradient directions and b-values used to acquire a diffusion-weighted dataset. A) Procedure step 1 - selection of the data for the lower b-value = 1000s/mm^2 which showed to be less corrupted by motion misalignments; B) Procedure step 2 – generation of the first version of data templates based on the selected lower b-value data; C) Procedure step 3 – realignment of the lower b-value data using the preliminary version of templates; D) Procedure step 4 – generation of the final version of data templates using the predicted signals for all original data b-values and diffusion gradient directions from the diffusion tensor model fitted to the lower b-value aligned data.

Appendix C. Supplementary Figures for Regional White Matter dMRI age profiles.

Here we show dMRI age profiles for selected WM ROIs across the age for all participants in the Cam-CAN's dMRI cohort. Supplementary Fig. S4 includes the ROIs where NDI and/or MSK showed positive age rates in the younger sub-group (i.e. Anterior Limb of the Internal Capsule, External Capsule, and Uncinate Fasciculus) and representative ROIs with earliest FA/MSK/NDI declines (i.e. Anterior Corona Radiata and Superior Frontal-Occipital Fasciculus). For a better inspection of the anterior-posterior degeneration gradients in the Corpus Callosum, Supplementary Fig. S5 shows the dMR age profiles for the Genu, Body, and Splenium of the Corpus Callosum.

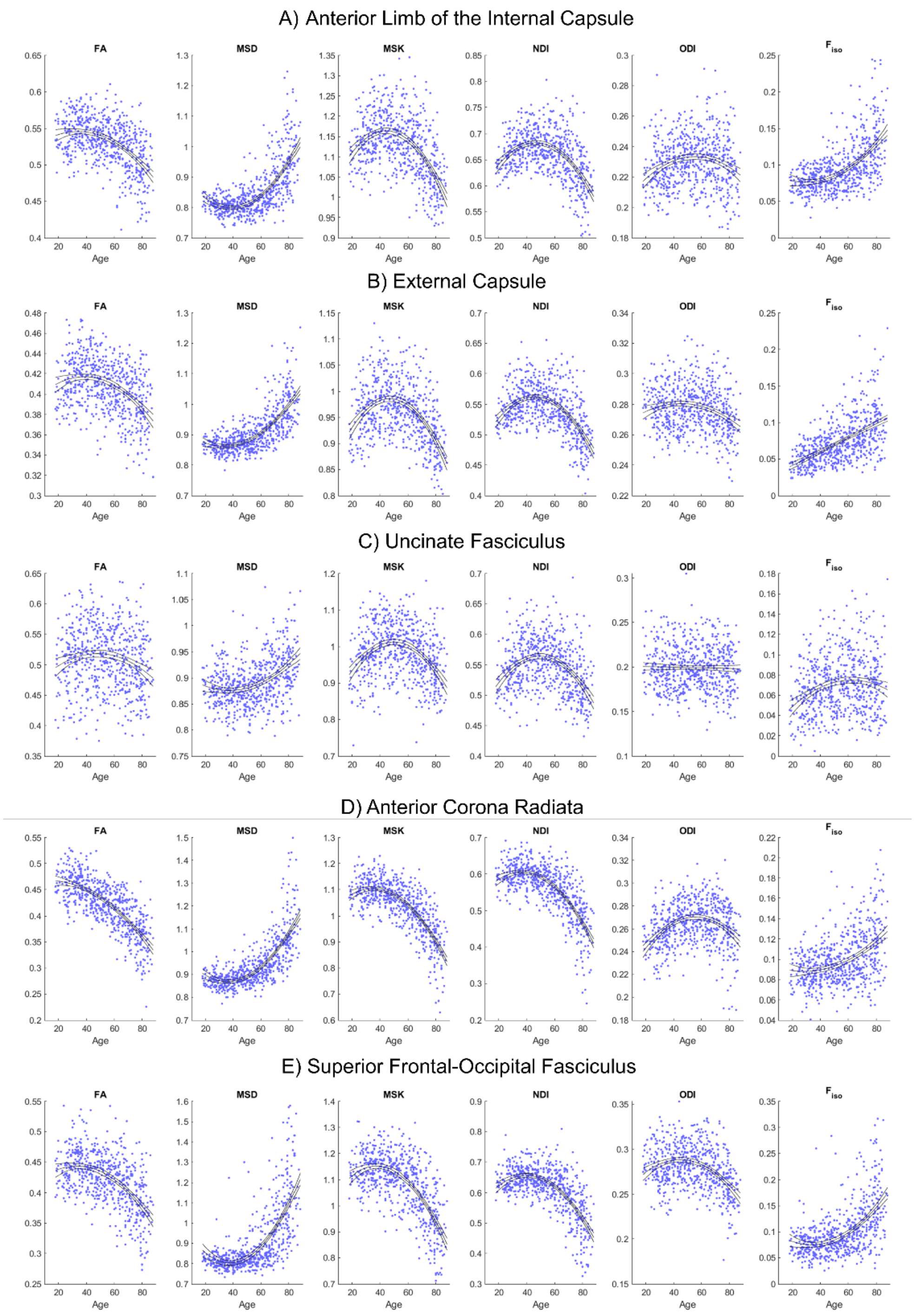

Supplementary Fig. S4 - Mean diffusion metrics extracted selected left-right averaged WM ROIs: A) Anterior Limb of the Internal Capsule; B) External Capsule; C) Uncinate Fasciculus; D) Anterior Corona Radiata; and E) Superior Frontal-Occipital Fasciculus.

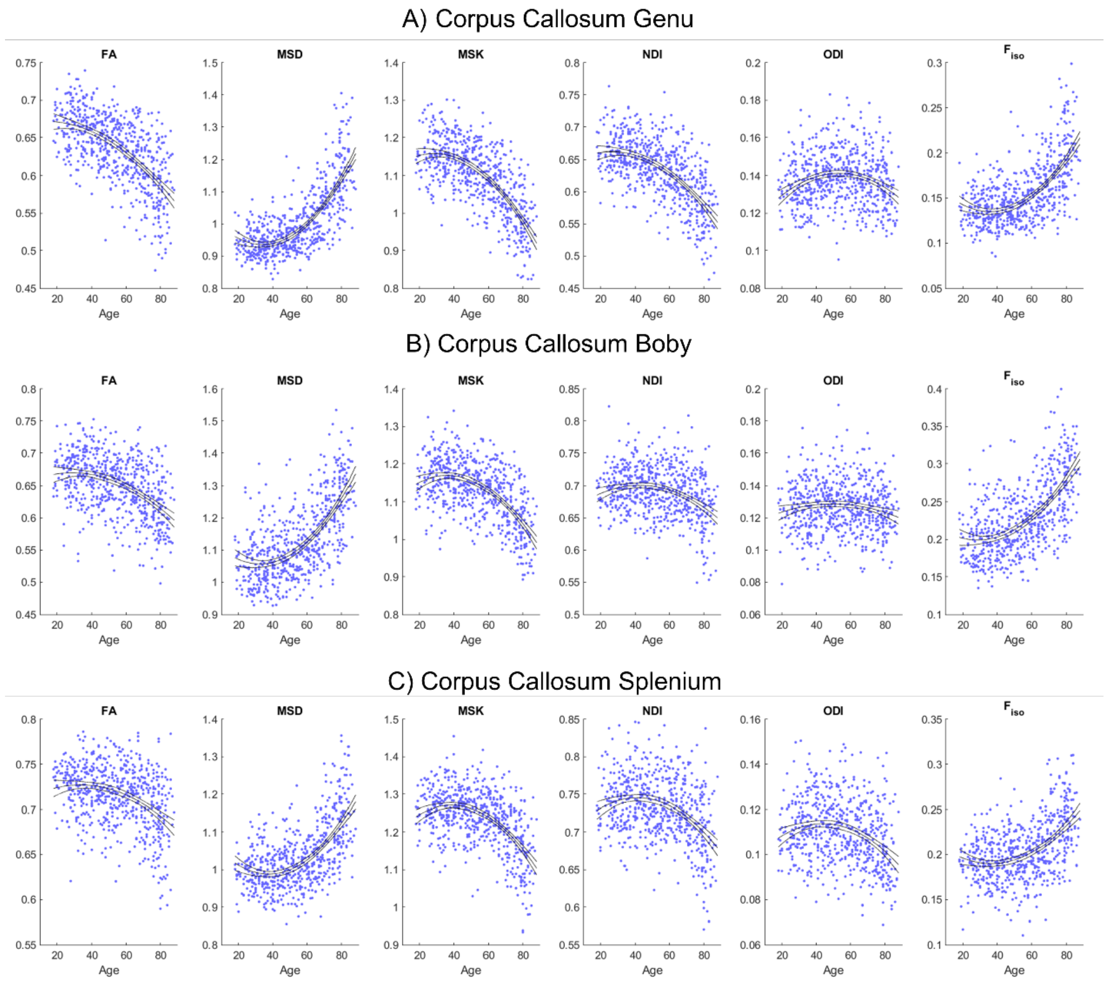

Supplementary Fig. S5 - Mean diffusion metrics extracted for Corpus Callosum WM ROIs: A) Corpus Callosum Genu; B) E Corpus Callosum Body; and C) Corpus Callosum Splenium.

Appendix D. Supplementary Results for Standard DKI Metrics

Additional results for the standard DKI metrics are reported in this supplementary material. Supplementary Fig. S6 shows representative maps for the standard DKI metrics (MD, RD, AD, MK, RK, and AK) for two young adults (26 and 25 years old, panels A and B) and for two elders (79 years old, panels C and D), while Supplementary Fig. S7 shows the loading of the factors when adding MD, RD, AD to the factor analysis and respective age profiles.

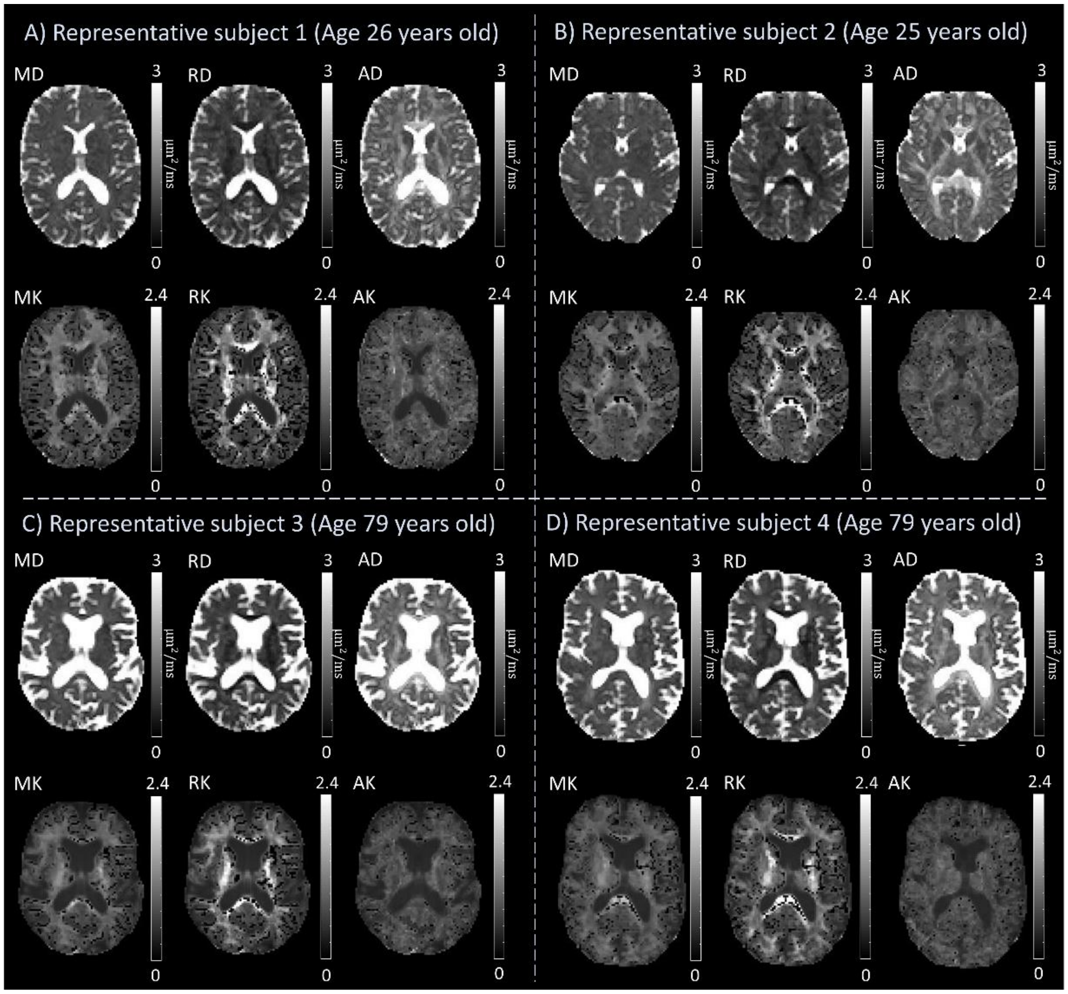

Supplementary Fig. S6 - Representative maps of standard DKI metrics (MD, RD, AD, MK, RK, and AK) for two young adults (26 and 25 years old, panels A and B) and for two elders (79 years old, panels C and D).

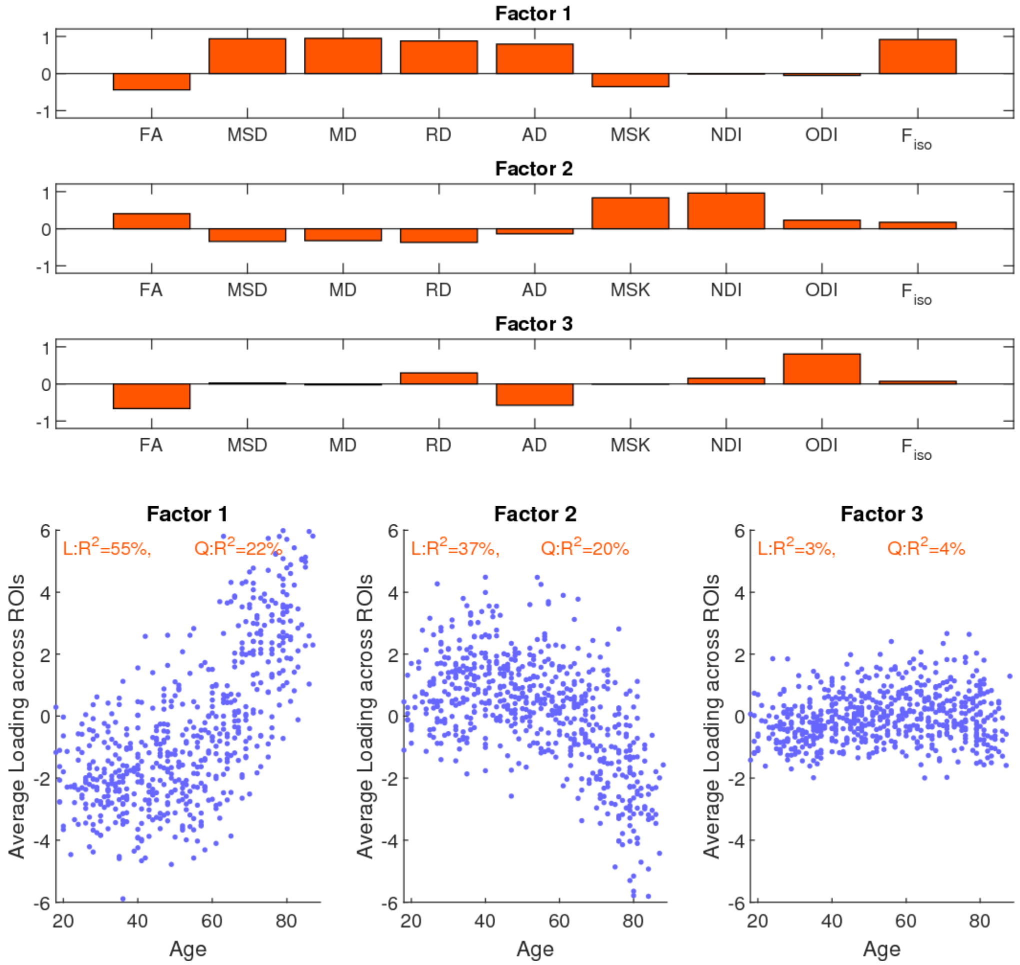

Supplementary Fig. S7 - Loadings of three factors from factor analysis across nice diffusion metrics (upper three panels) and their profiles against age (lower three panels).
